# Short-Term Laboratory Assessment of Coagulation-Assisted Ceramic Membrane Filtration and Reverse Osmosis Polishing of High-Strength Brewery Wastewater

**DOI:** 10.3390/membranes16070235

**Published:** 2026-07-08

**Authors:** Agnieszka Urbanowska, Izabela Polowczyk, Mateusz Kruszelnicki, Przemysław Seruga, Natalia Matura

**Affiliations:** 1 Department of Water and Wastewater Management and Waste Technology, Faculty of Environmental Engineering, Wrocław University of Science and Technology, Wybrzeże Wyspiańskiego 27, 50-370 Wrocław, Poland; 2 Department of Process Engineering and Technology of Polymer and Carbon Materials, Faculty of Chemistry, Wrocław University of Science and Technology, C. K. Norwida 4/6, 50-373 Wrocław, Poland; 3 Department of Bioprocess Engineering, Faculty of Production Engineering, Wrocław University of Economics and Business, Komandorska 118/120, 53-345 Wrocław, Poland

**Keywords:** brewery wastewater, membrane processes, ceramic membranes, coagulation, reverse osmosis, permeate quality

## Abstract

Brewery wastewater is a high-strength industrial effluent containing substantial organic, suspended, and colloidal fractions and therefore requires multistage treatment. This study evaluated sedimentation, prefiltration, coagulation, ceramic membrane filtration, and reverse osmosis (RO) polishing for improving the quality of actual brewery wastewater under short-term laboratory conditions. The acidic wastewater had chemical oxygen demand (COD), biochemical oxygen demand (BOD_5_), and dissolved organic carbon (DOC) values of 48,230 mg O_2_/L, 34,160 mg O_2_/L, and 6492 mg C/L, respectively. Three configurations were investigated: mechanical treatment; PIX 113 coagulation followed by ceramic microfiltration (MF), ultrafiltration (UF), or fine UF; and an integrated UF-RO system. Performance was assessed using contaminant removal, relative permeate flux (J/J_0_), particle size analysis, dynamic light scattering, and zeta potential. Sedimentation and prefiltration provided limited treatment, whereas coagulation effectively destabilized colloids; a PIX 113 dosage of 2 mL/L was selected as a favorable compromise among the tested dosages. Among the ceramic membrane-based trains, the train ending with the 1 kDa membrane produced the highest-quality permeate, with overall COD, BOD_5_, and DOC removals of 78.2%, 88.7%, and 49.8%, respectively. The tested sedimentation–prefiltration–coagulation-50 kDa UF-RO train achieved the highest overall removals: 97.9% COD, 98.6% BOD_5_, and 94.0% DOC. The overall removals of chloride and nitrate ions in this train were 92.5% and 68.5%, respectively. The results indicate that coagulation-assisted ceramic membrane filtration followed by RO can substantially improve permeate quality. The novelty of the work lies in linking coagulation-assisted ceramic membrane filtration and RO polishing with particle-size and electrokinetic characterization, thereby clarifying the role of each treatment barrier and identifying an effective laboratory-scale train for upgrading high-strength brewery wastewater.

## 1. Introduction

The brewing industry is among the most water-intensive sectors of the food industry and generates substantial volumes of wastewater. Water is used both as a product ingredient and as a processing and cleaning medium; therefore, its use and subsequent management are important to the environmental and operational efficiency of a brewery [1,2]. Brewery wastewater is generated at numerous stages of the production process, including brewing, fermentation, filtration, bottling, and equipment cleaning. Its volume and composition depend on the type and scale of production, the technology employed, and the brewery’s water and wastewater management practices. Brewery wastewater typically contains high concentrations of suspended solids, organic matter, and nutrients, while also exhibiting substantial variability in quality [3,4]. This variability complicates the selection of a treatment solution capable of providing both high treatment efficiency and stable operation under fluctuating loads [1].

From the perspective of industrial water and wastewater management, it is particularly important not only to reduce the pollutant load discharged with wastewater but also to improve the quality of the treated stream sufficiently to allow its further use to be considered. In water-intensive sectors such as brewing, wastewater treatment can form part of a strategy to reduce process water consumption, decrease the burden on sewer systems, and alleviate pressure on local water resources [4,5]. In this context, treatment trains must be developed that not only meet pollutant removal requirements but also produce a stream of sufficient quality for potential reuse or further polishing. This perspective allows membrane processes to be analyzed primarily as tools for improving wastewater quality and facilitating water recovery [6,7], without implying a comprehensive environmental or economic assessment of the entire system.

The complex nature of brewery wastewater results from the simultaneous presence of readily settleable fractions, fine suspended solids, stable colloidal systems, and dissolved substances. These contaminant groups differ in their susceptibility to removal; therefore, effective treatment of this type of wastewater generally requires multistage systems [4,5]. Simple mechanical processes can partially reduce suspended solids, but their effectiveness in removing stable colloids and dissolved compounds is insufficient [5,8]. Conversely, the direct application of advanced separation methods without adequate feed pretreatment may adversely affect short-term flux behavior, increase filtration resistance, and cause a rapid decline in process performance. The appropriate integration of pretreatment and advanced separation stages is therefore crucial to the quality of the resulting effluent and the practical implementation of the technology in industry [8,9].

Pressure-driven membrane processes play an important role among the methods available for the advanced treatment of brewery wastewater. Microfiltration (MF) and ultrafiltration (UF) enable the removal of larger suspended particles and colloids, whereas nanofiltration (NF) and reverse osmosis (RO) extend separation to selected dissolved constituents [10,11]. Membrane processes can therefore be used not only to reduce pollutant loads but also to improve the quality of the treated stream with a view to its potential recovery. Another advantage is the possibility of designing integrated systems that include both wastewater pretreatment and final membrane separation stages [7,8]. For brewery wastewater, however, a single membrane process generally does not provide sufficient treatment efficiency or favorable short-term flux behavior. These limitations primarily result from the presence of stable colloids and dissolved fractions, as well as organic compounds that promote membrane fouling [6].

Ceramic membranes are of particular interest because, compared with polymeric membranes, they exhibit greater chemical, thermal, and mechanical resistance as well as high operational durability [12,13]. These properties are important in the treatment of industrial wastewater with variable composition, high contaminant loads, and substantial membrane-fouling potential. Ceramic membranes may be particularly useful in systems that require stable operation, resistance to challenging operating conditions, and the ability to withstand rigorous cleaning [12,14]. From the perspective of industrial water and wastewater management, their use is especially relevant when it provides consistent permeate quality and reduces the risk of unstable system operation. However, the suitability of membrane processes is not determined solely by the membrane material. Feed characteristics, pretreatment methods, and the appropriate selection of successive treatment stages are equally important [9,15].

The relationships among the initial destabilization of the colloidal system, suspended solids removal, and the efficiency of subsequent membrane filtration remain insufficiently understood in the context of improving water management at industrial facilities [7,15]. In particular, it is important to determine the extent to which pretreatment processes, such as sedimentation, prefiltration, and coagulation, affect membrane process performance and the final quality of the resulting permeate [8,15]. From a practical perspective, a treatment configuration must be identified that effectively removes contaminants present in suspended, colloidal, and dissolved forms while producing a treated stream of a quality relevant to its subsequent management. It is also important to identify which process stages remove larger particle fractions, which reduce membrane fouling, and which are necessary to remove more persistent constituents that impair the quality of the treated stream [5,6].

Unlike earlier studies that focused on membrane-based recovery from brewery wastewater or broadly examined ceramic membranes and pretreatment-related fouling [6,12,15], this study comprehensively assesses multistage brewery wastewater treatment systems combining different feed pretreatment stages with advanced membrane separation, with particular emphasis on ceramic membranes. An important aspect of the study is the relationship established between treatment efficiency and the properties of the colloidal system, which provides a better understanding of the role of individual process stages in improving wastewater quality and reducing the load on subsequent separation stages. This approach enables not only a comparison of the investigated treatment configurations but also an assessment of their suitability for designing treatment systems aimed at improving effluent quality and increasing the potential for water recovery in the brewing industry. The study considers both the final quality of the resulting permeate and the function of individual unit operations in the sequential removal of contaminant fractions.

The aim of this study was to assess, under short-term laboratory conditions, the effectiveness of multistage treatment trains combining sedimentation, prefiltration, coagulation, ceramic membrane filtration, and RO polishing for improving the quality of high-strength brewery wastewater. Particular attention was given to the role of ceramic membranes in reducing suspended, colloidal, and macromolecular fractions after coagulation-assisted pretreatment. An additional objective was to evaluate the quality of the obtained permeates as a basis for further treatment or future reuse assessment. By combining treatment-efficiency data with particle size, DLS, and zeta-potential analyses, the study provides mechanistic insight into how pretreatment and ceramic membrane tightness shape permeate quality before RO polishing.

This approach is significant because it supports the rational design of multistage treatment trains in which mechanical pretreatment, coagulation, ceramic membrane filtration, and RO perform complementary functions. The findings help identify which stages are responsible for removing suspended, colloidal, macromolecular, and dissolved fractions, thereby strengthening the basis for future water recovery-oriented treatment strategies in the brewing industry.

## 2. Materials and Methods

### 2.1. Wastewater Sample

Actual brewery wastewater was collected from a craft brewery located in the Lower Silesian Voivodeship, Poland. The wastewater represented a mixed stream generated during the overall beer production process, rather than wastewater from a single unit operation. Several wastewater batches were collected and analyzed, and the values presented in Table 4 and in the Results and Discussion section are reported as mean values for the investigated batches. After collection, the samples were stored under refrigerated conditions until the experiments to limit changes in wastewater composition [16,17]. Before use, the wastewater was mixed manually to reduce sample heterogeneity.

### 2.2. Reagents and Membranes

Commercial PIX 113 coagulant supplied by Kemipol (Police, Poland) was used for coagulation. PIX 113 is an inorganic iron-based coagulant containing iron(III) sulfate. According to the product specifications, PIX 113 is a dark brown acidic liquid containing approximately 11.8% total iron. The coagulant density was approximately 1.50–1.58 g/cm^3^, and its pH was approximately 1 [18,19,20].

Flat-sheet ceramic membranes supplied by TAMI Industries (Nyons, France) were used for MF, UF, and fine UF/NF. A polymeric RO membrane supplied by TriSep Corporation (Goleta, CA, USA) was also used. The molecular weight cutoff (MWCO) values and other characteristics of all membranes tested are presented in Table 1.

### 2.3. Wastewater Treatment Configurations

Three treatment configurations were investigated to assess the effects of successive feed pretreatment stages and membrane properties on brewery wastewater treatment efficiency. The configurations are summarized in Table 2.

The 50 kDa UF membrane was selected as the UF pretreatment stage before RO not because it was assumed to be the optimal UF membrane, but because it represented a relatively open ceramic UF barrier after coagulation-assisted pretreatment. The purpose of this configuration was to verify whether such a UF stage, aimed mainly at reducing suspended, colloidal, and macromolecular fractions, could provide a feed suitable for subsequent RO polishing. Tighter ceramic membranes, such as 15, 5, and 1 kDa, were evaluated separately to assess the effect of MWCO on permeate quality, but their permeates were not subjected to RO in this study. Therefore, the 50 kDa UF-RO train should be interpreted as one tested integrated configuration rather than as an optimized UF-RO system.

Abbreviated designations for the samples and treatment configurations are used throughout the remainder of the manuscript, including in the text and figures; therefore, they are compiled in Table 3 as a nomenclature reference for the Results section.

Configuration 1 was used to determine whether the simple combination of sedimentation and MF was sufficient to treat brewery wastewater. Configuration 2 enabled assessment of the role of pretreatment and the effects of ceramic membrane MWCO or pore size on separation efficiency. Configuration 3 was used to assess whether high-quality permeate could be obtained using an integrated system comprising pretreatment, 50 kDa ceramic UF, and final RO polishing.

### 2.4. Sedimentation, Prefiltration, and Coagulation

In the first stage, the raw brewery wastewater was subjected to sedimentation to assess the settleability of suspended solids and initially reduce the load of larger particles. Sedimentation was conducted for 60 min, and samples were collected at 10 min intervals for COD, BOD_5_, and DOC analyses. Chloride and nitrate were also determined in the wastewater after sedimentation.

Subsequently, the wastewater after sedimentation was prefiltered through a ChemTech (Gliwice, Poland) polyester felt filter with a pore size of 1 µm. The resulting stream served as the feed for coagulation. To select the coagulant dosage, coagulation tests were performed using PIX 113 at dosages of 1, 2, and 4 mL/L of wastewater [26]. A measured volume of wastewater was introduced into 1 L reactors, followed by the appropriate volume of coagulant. The process was conducted in two stages: rapid mixing for 1 min at approximately 150 rpm, followed by slow mixing for 20 min at approximately 20 rpm. After 30 min settling, the supernatant was filtered by gravity through qualitative medium-speed cellulose filter paper with a diameter of 150 mm (catalogue no. 06-00A 102.150, Chemland, Stargard, Poland). This filter paper filtration step was used as a laboratory clarification step to remove easily separable flocs remaining after coagulation before subsequent membrane filtration. Consequently, the samples denoted as C1, C2, and C4 should be interpreted as the products of the combined sequence of sedimentation, 1 µm prefiltration, coagulation, post-coagulation settling, and qualitative filter paper filtration, rather than as the effect of coagulation alone.

Considering the declared total iron content of PIX 113 and its density, the tested dosages of 1, 2, and 4 mL/L corresponded approximately to 177–186, 354–373, and 708–746 mg Fe/L, respectively. Because these were relatively high coagulant dosages, the selected 2 mL/L dose was treated only as a laboratory working dose for preparing the feed for membrane filtration.

Turbidity, COD, BOD_5_, DOC, chloride, and nitrate were determined in the samples after coagulation. Based on the results, a PIX 113 dosage of 2 mL/L of wastewater was selected for further membrane filtration experiments as a working dosage providing a favorable compromise between organic contaminant removal and the amount of coagulant added. This dosage should not be interpreted as a globally optimal coagulation dose, but as the selected dose among the three dosages tested in this laboratory study. The sample obtained after coagulation, sedimentation, and filtration served as the feed for the subsequent MF, UF, and NF processes and, in the integrated configuration, for the UF + RO system.

### 2.5. Pressure-Driven Membrane Filtration

**Figure 1:**
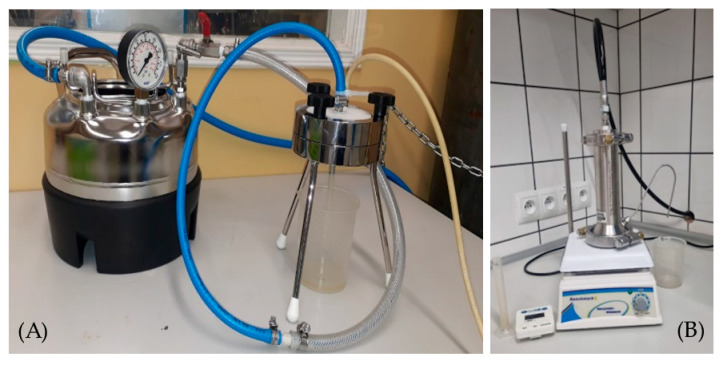
Filtration systems: (**A**) Sterlitech laboratory system with ceramic membranes; (**B**) Sterlitech high-pressure cell.

Pressure-driven membrane filtration was conducted in dead-end mode using two laboratory-scale membrane systems. Processes involving ceramic membranes were performed in a 3.80 L pressure cell (Sterlitech Corporation, Auburn, WA, USA) designed for flat-sheet ceramic membranes (Figure 1A). MF, UF, and fine UF/NF were performed at a transmembrane pressure of Δp = 0.3 MPa. Before the filtration experiments, the ceramic membranes were conditioned by filtering redistilled water at pressures ranging from 0.05 to 0.35 MPa until stable permeate flux values were obtained.

RO was performed using a Sterlitech HP4750 system designed for flat-sheet polymeric membranes (Figure 1B). The X201 RO membrane was conditioned at pressures ranging from 1 to 4 MPa. The RO process was conducted at a transmembrane pressure of Δp = 4 MPa using the permeate obtained after 50 kDa ceramic UF as the feed.

During filtration, the volume of permeate obtained was recorded as a function of time, enabling the permeate flux to be determined and changes in membrane permeability during the process to be assessed. In Configuration 1, wastewater after sedimentation was filtered using the 0.45 µm MF ceramic membrane. In Configuration 2, wastewater after sedimentation, prefiltration, and coagulation was divided into six homogeneous portions, which were then filtered using the following ceramic membranes: MF 0.45 µm, MF 0.14 µm, UF 50 kDa, UF 15 kDa, 5 kDa, and 1 kDa. In Configuration 3, the permeate obtained after 50 kDa UF was subjected to final polishing by RO. This variant was designed to test whether a relatively open UF membrane, used after sedimentation, prefiltration, and coagulation, could serve as a sufficient pretreatment stage before RO. The configuration was not intended to identify the optimal UF membrane for RO pretreatment, because RO was not tested with permeates obtained from the other ceramic UF membranes.

### 2.6. Membrane Preparation and Regeneration

Before the experiments, all flat-sheet ceramic membranes were prepared for operation. The procedure comprised sequential alkaline cleaning, rinsing with redistilled water, acid cleaning, and a final rinse until a neutral pH was obtained. First, the membranes were immersed for 30 min in a 15–20 g/L NaOH solution maintained at 80 °C. They were then rinsed with redistilled water until a neutral pH was obtained.

The acid-cleaning conditions depended on the type of ceramic membrane. For the MF and UF membranes, either 58% HNO_3_ or 75% H_3_PO_4_ was added at a dosage of 5 mL of acid per liter of cleaning solution. Cleaning was conducted at 50 °C for 15 min. For the NF membranes, only 75% H_3_PO_4_ was used, in accordance with the manufacturer’s recommendations, at a dosage of 1 mL of acid per liter of cleaning solution; cleaning was also conducted at 50 °C for 15 min. After acid cleaning, the membranes were rinsed again with redistilled water until a neutral pH was obtained.

A flat-sheet polymeric RO membrane was used in the final polishing stage. The X201 membrane supplied by TriSep Corp. was a thin-film composite RO membrane with an aromatic polyamide/polyamide-urea active layer. According to the membrane specifications, it is intended for industrial and wastewater applications, has a recommended pH operating range of 2–11, a maximum operating temperature of 45 °C, and a maximum operating pressure of 4.0 MPa. The active membrane area in the HP4750 cell was 17.4 cm^2^.

Before the RO experiment, the membrane was conditioned separately from the ceramic membranes. Conditioning consisted of filtering redistilled water in the Sterlitech HP4750 high-pressure cell at pressures increasing from 1 to 4 MPa until a stable water flux was obtained. The initial permeate obtained during conditioning was discarded. The RO process was then conducted at Δp = 4 MPa using the permeate obtained after 50 kDa ceramic UF as the feed.

The RO membrane was not subjected to the alkaline–acid preparation procedure used for ceramic membranes. In particular, the alkaline cleaning step with NaOH at elevated temperature and the subsequent acid-cleaning step were applied only to the ceramic MF, UF, and fine UF/NF membranes. This distinction is important because the X201 RO membrane contains a polyamide-based active layer and has a narrower allowable pH range than the ceramic membranes.

After filtration experiments, chemical regeneration with 0.1 mol/dm^3^ NaOH was applied only to the ceramic MF, UF, and fine UF/NF membranes. The ceramic membranes were then rinsed with redistilled water until neutral pH was obtained. This alkaline cleaning step was not applied to the X201 RO membrane because such a strongly alkaline solution exceeds the pH range specified for this membrane. After the RO experiment, the membrane was rinsed with redistilled water. Therefore, the reported RO performance should be interpreted as the performance of the conditioned X201 membrane during a short-term polishing experiment, not as the performance after repeated chemical regeneration cycles.

### 2.7. Analytical Methods

The effectiveness of the individual treatment stages was evaluated based on changes in selected physicochemical parameters. COD, BOD_5_, DOC, chloride, and nitrate were primarily determined in the raw wastewater, pretreated samples, and permeates. Physicochemical analyses were conducted using procedures commonly applied in water and wastewater testing and aligned with standard water and wastewater analytical methods [27].

COD was determined using the dichromate method [28], and BOD_5_ was determined using the dilution method [29]. DOC was determined by high-temperature oxidation in non-purgeable organic carbon (NPOC) mode using IL550 TOC-TN analyzer (Hach Lange GmbH, Düsseldorf, Germany) [30]. Inorganic ions, including chloride and nitrate, were determined by ion chromatography using a Dionex Aquion ion chromatograph (Thermo Fisher Scientific, San Jose, CA, USA) equipped with a conductivity detector for anion or cation analysis [31]. Depending on the scope of the analysis, this method also enabled the determination of fluoride, nitrite, bromide, phosphate, and sulfate. Sodium and potassium, when determined for a given sample series, were measured using a BWB-XP flame photometer (BWB Technologies Ltd., Newbury, UK).

In addition, pH and electrical conductivity were determined for selected samples. The pH was measured potentiometrically using an HQ40D digital multimeter equipped with an IntelliCAL™ PHC101 electrode (Hach Company, Loveland, CO, USA). Conductivity was measured using the conductometric method. For samples in which dissolved organic matter was assessed spectrophotometrically, UV absorbance at wavelengths of 254 and 272 nm was measured using a UV-VIS 1800 spectrophotometer (Shimadzu Corporation, Kyoto, Japan).

Particle size distribution and zeta potential analyses were performed to determine changes in the colloidal properties of the system. The particle size distribution of samples collected before membrane filtration was determined by laser diffraction [32] using a LS 13 320 XR analyzer (Beckman Coulter, Inc., Brea, CA, USA). The measurement range of the instrument was 10 nm–3500 µm. Measurements were performed using a liquid dispersion unit by adding sufficient sample to the circulating water to obtain the recommended signal level. The results were reported as D10, D50, and D90 values. Each analysis was performed in triplicate.

Membrane permeates with sufficiently low turbidity were characterized by dynamic light scattering (DLS) [33] using a Zetasizer Ultra RED analyzer (Malvern Panalytical Ltd., Malvern, UK). The instrument enabled measurements at detection angles of 13°, 90°, and 173°. Backscatter detection at 173°, based on non-invasive backscatter (NIBS) technology, was used for data interpretation because it provided scattering intensities within the optimal measurement range for the samples tested. The mean hydrodynamic diameter and polydispersity index (PDI) were determined for each sample. Each sample was analyzed in triplicate.

Zeta potential was determined using a Zetasizer Ultra RED analyzer and electrophoretic light scattering (ELS) [34]. Samples were placed in disposable DTS1070 capillary cells (Malvern Panalytical Ltd., Malvern, UK). Three measurements were performed for each sample in the automatic data-analysis mode, in which the software selected the calculation model based on sample conductivity. Each measurement comprised 10–30 sub-runs, with a 5 s interval between consecutive measurements. All particle size and zeta potential measurements were performed at 22 °C.

Each treatment experiment, including sedimentation, prefiltration, coagulation, membrane filtration, and RO, was performed in duplicate, comprising the main experiment and one independent repetition conducted under the same operating conditions. The values presented in the figures and discussed in the text are reported as mean values from duplicate experiments. Analytical determinations were performed according to the relevant analytical procedures, and instrumental measurements of particle size distribution, DLS, and zeta potential were carried out in triplicate for each analyzed sample. Because the number of independent process repetitions was limited, the results were interpreted as trends observed under short-term laboratory conditions, and no formal statistical significance analysis was performed. Where error bars are shown, they represent the range between duplicate independent experiments.

### 2.8. Performance Parameters and Calculations

The transport properties of the membranes were evaluated based on the volumetric permeate flux determined for both redistilled water and brewery wastewater [35,36]. The permeate flux was calculated using the following equation:

$$J=\frac{V}{A\cdot t}$$

where:

J—volumetric permeate flux, m^3^/(m^2^·d), V—permeate volume, m^3^, A—active membrane area, m^2^, and t—filtration time, d.

The relative permeate flux was used to assess the extent of membrane fouling and was calculated as the ratio of the permeate flux obtained during wastewater filtration to the redistilled water flux for the same membrane:

$$\frac{J}{J_{0}}$$

where: J—permeate flux during filtration of the sample, J_0_—redistilled water flux for the conditioned membrane.

Separation efficiency was evaluated using two complementary indices: overall removal and stage-specific removal. Overall removal was calculated relative to the raw brewery wastewater and was used to describe the cumulative effect of the successive treatment stages:

$$R_{overall}=(1-c_{t}/c_{raw})\times 100\%$$

where R_overall_ is the overall removal, c_t_ is the concentration after a given treatment stage or in the final permeate, and c_raw_ is the concentration in the raw wastewater.

**Figure 2:**
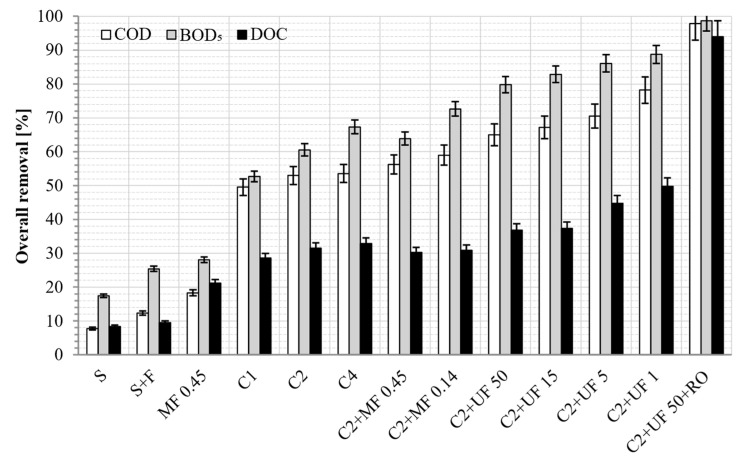
Overall removal of organic contaminants after successive stages of brewery wastewater treatment.

**Figure 3:**
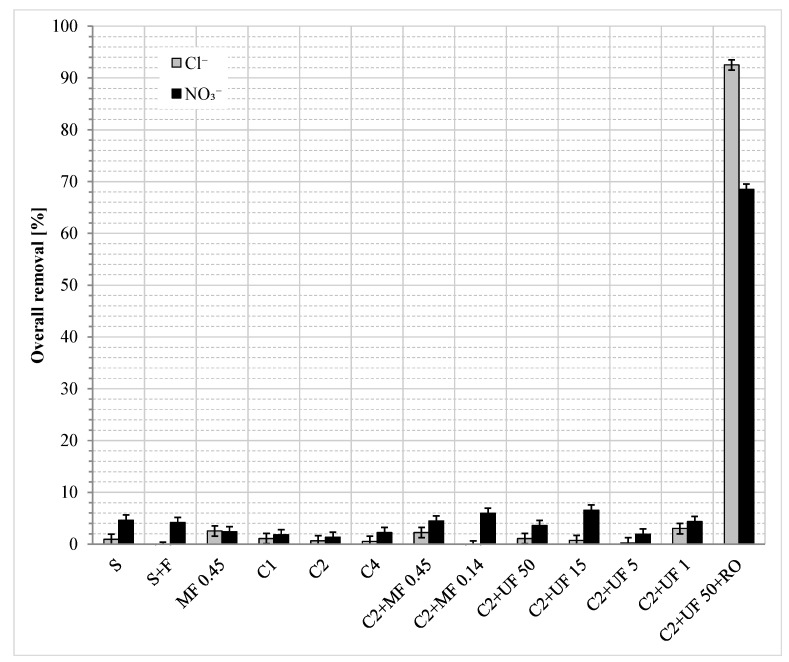
Overall removal of chloride (Cl^−^) and nitrate (NO_3_^−^) ions after successive stages of brewery wastewater treatment.

Stage-specific removal, referred to as observed membrane rejection for membrane stages, was calculated relative to the direct feed entering a given stage:

$$R_{stage}=(1-c_{p}/c_{f})\times 100\%$$

where R_stage_ is the removal in a given treatment stage, c_p_ is the concentration in the permeate or treated stream after this stage, and c_f_ is the concentration in the direct feed to this stage. Unless otherwise stated, the values shown in Figure 2 and Figure 3 represent overall removal calculated relative to the raw wastewater. Stage-specific values are discussed separately where the contribution of individual membrane stages is evaluated.

## 3. Results and Discussion

The results should be interpreted as a short-term laboratory assessment of the investigated treatment configurations. Because each process was performed in duplicate, the discussion focuses on the main observed trends and on differences that were consistent between repeated experiments. Minor differences between individual membranes or coagulant dosages should therefore be interpreted with caution and should not be regarded as statistically robust effects.

### 3.1. Changes in Organic Contaminant Content During Multistage Brewery Wastewater Treatment

The values in Table 4 should therefore be interpreted as average characteristics of the mixed brewery wastewater batches investigated in this study. Although this sampling approach better reflects the combined wastewater generated during beer production than a single process-specific sample, the results remain specific to the investigated brewery and laboratory conditions.

The wastewater was acidic and had a very high organic load, as indicated by its high chemical oxygen demand (COD), biochemical oxygen demand (BOD_5_), and dissolved organic carbon (DOC) values. The relatively high suspended solids content and the particle size distribution confirmed the presence of suspended and colloidal fractions. Because the wastewater contained suspended solids, colloidal fractions, and dissolved substances, it was treated using configurations comprising sedimentation, prefiltration, coagulation, and pressure-driven membrane processes. Dissolved solids were calculated as the difference between dry residue/total solids and suspended solids. The sum of mineral and volatile solids corresponded closely to the dry residue, with minor differences resulting from analytical uncertainty and rounding. The relatively moderate conductivity, despite the high dry residue, reflected the predominance of volatile/organic solids over mineral ionic constituents.

When assessing the suitability of the individual technological configurations for brewery wastewater treatment, particular attention was given to changes in the parameters describing organic matter content, namely COD, BOD_5_, and DOC. Because the presented values are mean values obtained for several mixed wastewater batches, the discussion focuses on general trends observed for the investigated brewery wastewater rather than on the behavior of a single grab sample. Figure 2 presents overall organic contaminant removal calculated relative to the raw wastewater; these cumulative values strongly depended on the treatment stage applied and the tightness of the membrane used for final separation. The raw brewery wastewater had a very high organic load, with COD, BOD_5_, and DOC values of 48,230 mg O_2_/L, 34,160 mg O_2_/L, and 6492 mg C/L, respectively. The relatively high BOD_5_/COD ratio of approximately 0.71 indicated a substantial proportion of biodegradable compounds; however, the very high absolute values of the analyzed parameters indicated the need for a multistage treatment system.

Sedimentation was the first treatment stage and was intended to reduce the readily settleable fraction. After 30 min of sedimentation, COD decreased from 48,230 to 44,500 mg O_2_/L, corresponding to a removal of 7.7%. The decrease in BOD_5_ was more pronounced, reaching 17.4%, whereas DOC decreased by only 8.3%. These results indicate that sedimentation primarily removed suspended and readily settleable matter containing a substantial proportion of biodegradable substances. The lower DOC removal indicates that a considerable proportion of organic carbon remained in the system in dissolved or colloidal form and was therefore not amenable to simple gravity separation.

Additional prefiltration through a 1 µm filter further improved wastewater quality, although the extent of this improvement was limited. Following the combination of sedimentation and prefiltration, COD, BOD_5_, and DOC were 42,300 mg O_2_/L, 25,480 mg O_2_/L, and 5872 mg C/L, respectively, corresponding to overall removals from the raw wastewater of 12.3%, 25.4%, and 9.6%. These results indicate that the mechanical removal of larger particles is insufficient for effective brewery wastewater treatment. The particularly low DOC removal after this stage indicates the presence of a substantial quantity of organic compounds in dissolved or fine-colloidal form or in stable dispersions.

MF using the 0.45 µm ceramic membrane after sedimentation achieved greater reductions in the analyzed parameters than sedimentation and prefiltration alone. COD decreased to 39,400 mg O_2_/L, BOD_5_ to 24,560 mg O_2_/L, and DOC to 5118 mg C/L. These values corresponded to removals from the raw wastewater of 18.3%, 28.1%, and 21.2%, respectively. The results indicate that a simple combination of sedimentation and MF can remove some fine suspended solids and colloidal matter but does not sufficiently reduce the organic contaminant load. This configuration can therefore be regarded as a preliminary wastewater clarification stage rather than a stand-alone treatment method capable of producing a stream of suitable quality for further use.

Substantially greater treatment efficiency was achieved using PIX 113 coagulation. At a dosage of 1 mL PIX 113/L of wastewater, COD, BOD_5_, and DOC decreased to 24,350 mg O_2_/L, 16,160 mg O_2_/L, and 4638 mg C/L, respectively. Increasing the coagulant dosage to 2 mL PIX 113/L of wastewater further decreased these values to 22,680 mg O_2_/L, 13,480 mg O_2_/L, and 4445 mg C/L, corresponding to removals from the raw wastewater of 53.0%, 60.5%, and 31.5%, respectively. At the highest dosage of 4 mL PIX 113/L of wastewater, COD, BOD_5_, and DOC values of 22,400 mg O_2_/L, 11,160 mg O_2_/L, and 4359 mg C/L, respectively, were obtained. Increasing the coagulant dosage from 2 to 4 mL PIX 113/L of wastewater therefore produced only a slight improvement in COD and DOC removal, whereas a more pronounced effect was observed for BOD_5_. This may indicate that at 2 mL PIX 113/L of wastewater, the colloidal fraction had already been substantially destabilized and most of the contaminants contributing to COD had been removed, whereas further increasing the dosage primarily affected removal of the more biodegradable fraction. From a technological perspective, a dosage of 2 mL PIX 113/L of wastewater provided a favorable compromise between treatment efficiency and the amount of coagulant added.

The results obtained for wastewater subjected to coagulation and membrane filtration indicate that proper membrane selection is important to the final permeate quality. MF after coagulation produced only a moderate improvement in wastewater quality. With the 0.45 µm MF membrane, COD, BOD_5_, and DOC were 21,100 mg O_2_/L, 12,340 mg O_2_/L, and 4528 mg C/L, respectively, whereas with the 0.14 µm MF membrane, the respective values were 19,780 mg O_2_/L, 9340 mg O_2_/L, and 4488 mg C/L. Reducing the membrane pore size from 0.45 to 0.14 µm primarily improved BOD_5_ removal, indicating more effective separation of the biodegradable fine suspended and colloidal fractions. At the same time, changes in DOC were minor, and no clear improvement over the sample subjected to coagulation alone was observed. Thus, even after prior destabilization of the colloidal system, MF does not provide a sufficient barrier to dissolved organic compounds.

A more pronounced improvement in wastewater quality was achieved using UF membranes. For the 50 kDa UF membrane, COD, BOD_5_, and DOC were 16,890 mg O_2_/L, 6900 mg O_2_/L, and 4098 mg C/L, respectively. The tighter 15 kDa UF membrane reduced these values to 15,820 mg O_2_/L, 5850 mg O_2_/L, and 4066 mg C/L. Further decreasing the MWCO to 5 kDa resulted in COD, BOD_5_, and DOC values of 14,230 mg O_2_/L, 4750 mg O_2_/L, and 3584 mg C/L, respectively. Among the ceramic membranes applied after coagulation, the most favorable results under the tested conditions were obtained with the 1 kDa membrane, for which the analyzed parameters were 10,500 mg O_2_/L, 3850 mg O_2_/L, and 3260 mg C/L. These values corresponded to overall removals from the raw wastewater of 78.2% for COD, 88.7% for BOD_5_, and 49.8% for DOC.

Because these overall removals include sedimentation, prefiltration, coagulation, post-coagulation settling, filter paper filtration, and membrane filtration, they should not be interpreted as the rejection of the individual ceramic membrane alone. To separate the contribution of the membrane stage, observed membrane rejection was also calculated relative to the C2 feed entering each membrane stage (Table 5).

The stage-specific values show that the ceramic membranes provided additional removal after C2 pretreatment, but the magnitude of this effect was lower than the overall removals shown in Figure 2. The DOC rejection values reported as approximately zero for both MF membranes indicate no measurable DOC rejection within the uncertainty of the experiment, most probably reflecting analytical variability, differences between subsamples, or changes in the sample during the experiment rather than actual DOC release by the membranes.

The observed relationship between membrane MWCO and permeate quality was consistent with separation in the investigated system being governed by both size exclusion and prior destabilization of the colloidal fraction during coagulation. As membrane tightness increased, a greater proportion of particles and organic macromolecules was retained. This effect was particularly evident for BOD_5_, whose removal increased as membrane MWCO decreased. This indicates that the fraction contributing to BOD_5_ was largely associated with fine suspended solids, colloids, or macromolecules that could be effectively removed by coagulation and UF. Conversely, the lower DOC removal, even with the 1 kDa membrane, indicates the presence of a substantial fraction of low-molecular-weight dissolved organic compounds that passed through the ceramic MF, UF, and fine UF membranes.

The change in the relationship between COD and BOD_5_ during successive treatment stages is also noteworthy. The BOD_5_/COD ratio was approximately 0.71 in the raw wastewater but decreased to approximately 0.37 after treatment with the 1 kDa membrane. This indicates that the successive treatment stages removed the readily biodegradable fraction more effectively than the total load of chemically oxidizable compounds. Consequently, a substantial proportion of less biodegradable organic compounds or dissolved compounds that were retained to a lesser extent by UF remained in the permeate from the 1 kDa membrane.

The highest treatment efficiency among the tested configurations was achieved in the integrated system comprising sedimentation, prefiltration, coagulation, 50 kDa UF, and final RO polishing. After RO, COD, BOD_5_, and DOC were 1033 mg O_2_/L, 468 mg O_2_/L, and 389 mg C/L, respectively. These values corresponded to overall removals from the raw wastewater of 97.9%, 98.6%, and 94.0%, respectively. Compared with the permeate from the 50 kDa UF membrane, the RO stage reduced COD, BOD_5_, and DOC by 93.9%, 93.2%, and 90.5%, respectively. This substantial improvement in permeate quality indicates that RO was the key stage for removing the dissolved fraction that was not effectively eliminated by MF and UF.

The results demonstrate that the individual treatment stages served different functions. Sedimentation and prefiltration primarily reduced the readily settleable fraction and larger particles. Coagulation destabilized the colloidal system and substantially reduced COD and BOD_5_. The ceramic MF and UF membranes acted as barriers to particles, colloids, and organic macromolecules, with the effectiveness of this stage increasing as membrane MWCO decreased. RO, in turn, was essential for the extensive removal of dissolved organic compounds, particularly those contributing to DOC in the permeates from the preceding treatment stages.

From the perspective of designing brewery wastewater treatment systems, these results indicate that individual mechanical processes or MF alone do not provide sufficient organic contaminant removal. The system combining coagulation and fine UF with the 1 kDa ceramic membrane substantially reduced COD and BOD_5_ but still left a relatively high DOC concentration. If the objective is to obtain the highest possible quality of the treated stream for potential water recovery, a final polishing stage using a dense membrane is required. In the investigated system, this function was fulfilled by the final RO stage, which provided a large additional decrease in all analyzed organic parameters relative to the preceding 50 kDa UF permeate.

The results therefore support the use of multistage brewery wastewater treatment systems in which the individual unit operations perform complementary functions. Process efficiency depends not only on the properties of the final membrane but also on the sequential removal of suspended, colloidal, macromolecular, and dissolved contaminant fractions. This approach reduces the load on subsequent separation stages, improves permeate quality, and increases the potential for applying membrane processes in industrial wastewater treatment systems aimed at water recovery.

From a cost-effectiveness perspective, the treatment train should therefore be selected according to the required treatment endpoint rather than by adopting the most intensive configuration in all cases. If the treated stream is intended mainly for pretreatment before biological treatment, discharge to an external treatment plant, or reduction in suspended, colloidal, and biodegradable organic load, coagulation followed by ceramic UF may be more appropriate because it avoids the additional pressure, energy demand, membrane replacement, and concentrate management requirements associated with RO [5,6,37]. In such applications, the 1 kDa ceramic membrane represented the most effective non-RO option tested, whereas the 50 kDa UF stage should be interpreted primarily as the pretreatment used before RO in this study. RO is justified when the target application requires substantial removal of dissolved organic matter, residual color, and inorganic ions, for example when high-quality water recovery is considered. Thus, the results support a stepwise selection criterion: use the least intensive treatment train that meets the required discharge or reuse objective, and add RO only when MF/UF permeate quality is insufficient for that endpoint.

### 3.2. Overall Removal of Chloride and Nitrate Ions During Multistage Brewery Wastewater Treatment

In addition to organic contaminant removal, an important aspect of evaluating the multistage brewery wastewater treatment system was the analysis of changes in the concentrations of selected inorganic ions, namely chloride and nitrate. Figure 3 presents overall removal calculated relative to the raw wastewater. The cumulative removal of these ions was low during most of the treatment stages analyzed. This is consistent with the nature of the investigated processes because sedimentation, prefiltration, coagulation, MF, and UF primarily remove suspended solids, colloids, macromolecules, and some organic compounds but do not provide an effective barrier to small inorganic ions present in dissolved form.

For chloride ions, overall removal after sedimentation was only approximately 0.9%, whereas the combination of sedimentation and 1 µm prefiltration yielded a slightly negative value (−0.6%). Similarly low values were observed after coagulation, regardless of the PIX 113 dosage applied. Overall chloride removals at dosages of 1, 2, and 4 mL PIX 113/L of wastewater were approximately 1.1%, 0.6%, and 0.5%, respectively. Thus, despite its substantial effect on organic compound removal, coagulation did not effectively remove chloride ions. This is expected because chloride is present in wastewater as a highly soluble monovalent ion that does not form readily removable aggregates under conditions typical of brewery wastewater coagulation.

The use of ceramic MF and UF membranes after coagulation also did not substantially increase overall chloride removal. For the treatment trains ending with the 0.45 µm MF, 50 kDa UF, 15 kDa UF, and 5 kDa UF membranes, overall chloride removal remained between approximately 0.2% and 2.2%. The highest value among the ceramic membrane-based trains was obtained with the 1 kDa fine UF membrane; however, overall chloride removal was only approximately 3.0% in this case. These low values indicate that the separation mechanisms dominant in MF and UF, primarily size exclusion and the retention of particles and macromolecules, are insufficient for removing small inorganic ions. Values close to zero or slightly negative should be interpreted as an absence of measurable removal resulting from very small differences in concentration between the feed and permeate and from possible analytical variability.

A similar trend was observed for nitrate ions, although their overall removal during some treatment stages was slightly higher than that of chloride. After sedimentation, overall NO_3_^−^ removal was approximately 4.6%, whereas after sedimentation and prefiltration, it was approximately 4.2%. Following coagulation, these values remained low, ranging from approximately 1.3% to 2.2%. Applying ceramic membranes after coagulation produced only a slight improvement in overall nitrate removal. Among the systems without RO, the highest overall removal was obtained with the 15 kDa UF membrane, at approximately 6.6%, and the 0.14 µm MF membrane, at approximately 6.0%. However, these differences do not indicate a clear relationship between membrane MWCO and nitrate removal. This suggests that nitrate, like chloride, remained in the wastewater primarily in ionic form and passed through the MF and UF membranes with the permeate.

A distinctly different effect was achieved only in the treatment train ending with RO as the final treatment stage. In the system comprising sedimentation, prefiltration, coagulation, 50 kDa UF, and RO, overall chloride removal increased to approximately 92.5%, whereas overall nitrate removal increased to approximately 68.5%. These values describe the cumulative effect of the whole treatment train and should not be interpreted as isolated RO rejection values. The substantial difference between the MF/UF-based trains and the RO-ending train results from their different separation mechanisms. An RO membrane forms a dense separation barrier in which the transport of dissolved constituents is restricted not only by molecular or ionic size but also by the solution diffusion mechanism, electrostatic interactions, and the properties of the membrane active layer. Therefore, only the treatment train including RO enabled effective removal of inorganic ions present in dissolved form.

A comparison of chloride and nitrate overall removal in the RO-ending treatment train indicates that chloride concentration decreased more strongly than nitrate concentration. This difference may result from differences in ion mobility, hydration properties, interactions with the membrane active layer, and operating conditions. However, both ions analyzed are small monovalent ions whose removal is considerably more difficult using porous membranes than dense membranes. The results indicate that a final RO stage is crucial when the objective of brewery wastewater treatment is not only to reduce organic compounds but also to decrease the concentration of mineral constituents.

The results presented in Figure 3 complement the observations concerning organic contaminant removal. Sedimentation, prefiltration, coagulation, and MF/UF can effectively reduce suspended solids, colloids, and some macromolecular organic contaminants but do not substantially decrease the concentration of small inorganic ions. Consequently, a system based solely on coagulation and MF/UF membrane filtration may be sufficient for wastewater pretreatment or clarification but not as a complete polishing process aimed at reducing salinity. Introducing RO as the final treatment stage fundamentally changes the treatment outcome by enabling both extensive organic contaminant removal and substantial overall removal of dissolved ions.

From a technological perspective, the results indicate that the individual stages of the investigated system perform different functions. The pretreatment stages and ceramic MF/UF membranes primarily protect the RO membrane from excessive loading by suspended solids, colloids, and organic matter, whereas a substantial decrease in chloride and nitrate ion concentrations was observed only for the treatment train ending with RO. This supports the use of an integrated system in which pretreatment improves the operating conditions of the final membrane and RO determines permeate quality with respect to dissolved constituents.

### 3.3. Short-Term Changes in Relative Permeate Flux During Membrane Filtration

The analysis of membrane hydraulic behavior was based on short-term, 40 min dead-end filtration tests and on changes in relative permeate flux (J/J_0_). Therefore, the results should be interpreted as short-term flux behavior under laboratory conditions, not as evidence of long-term operational stability. Because resistance-in-series analysis, blocking model fitting, and membrane surface characterization were not performed, the discussion of fouling mechanisms is limited to possible explanations consistent with the observed J/J_0_ trends.

**Figure 4:**
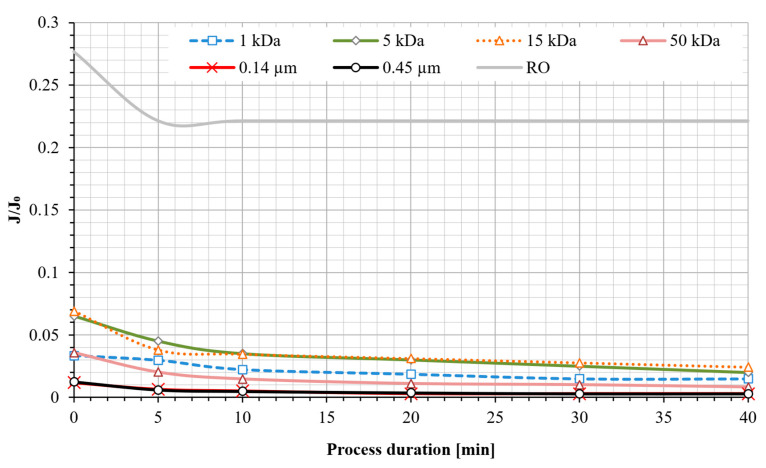
Changes in relative permeate flux (J/J_0_) as a function of filtration time for microfiltration (MF), ultrafiltration (UF), fine UF, and reverse osmosis (RO) membranes used in brewery wastewater treatment.

An assessment of brewery wastewater treatment efficiency should consider not only the quality of the resulting permeate but also short-term flux behavior. For this purpose, changes in relative permeate flux were analyzed and expressed as the ratio of the current permeate flux to the initial redistilled water flux for a given membrane (J/J_0_). This parameter enables comparison of membrane fouling susceptibility independently of absolute differences in hydraulic permeability. The results presented in Figure 4 indicate that all ceramic MF and UF membranes tested exhibited a substantial decrease in relative permeate flux from the beginning of the process, which was consistent with the high fouling potential of the brewery wastewater.

The lowest J/J_0_ values were observed for the 0.14 and 0.45 µm MF membranes. At the beginning of the process, the relative permeate flux was approximately 0.012 for both membranes and decreased to approximately 0.003 after 40 min of filtration. Thus, the actual permeate flux represented only a small fraction of the initial membrane flux measured using redistilled water. This pronounced decrease in relative permeability suggests a substantial increase in filtration resistance, which may have been related to the deposition or partial blockage caused by fine suspended solids, colloidal matter, and aggregates formed during the preceding treatment stages. Despite the larger pores of the MF membranes, the presence of particles similar in size to the pore diameter may have promoted pore constriction and/or the formation of a deposit layer on the membrane surface; however, these mechanisms cannot be distinguished on the basis of J/J_0_ data alone.

The UF and fine UF membranes produced higher J/J_0_ values than the MF membranes; however, their values also indicated a substantial increase in filtration resistance over time. Among the ceramic membranes, the highest initial relative permeate fluxes were observed for the 15 and 5 kDa membranes, with J/J_0_ values of approximately 0.069 and 0.065, respectively. After 40 min of filtration, these values decreased to approximately 0.024 for the 15 kDa membrane and 0.020 for the 5 kDa membrane. The 15 kDa membrane therefore exhibited the most favorable short-term flux behavior among the ceramic membranes tested, combining a relatively high initial J/J_0_ with the highest final value after 40 min of operation.

The 1 kDa membrane had a lower initial relative permeate flux than the 5 and 15 kDa membranes; however, the decrease in J/J_0_ over time was less pronounced than for the 50 kDa membrane and the MF membranes. J/J_0_ decreased from approximately 0.033 at the beginning of filtration to approximately 0.015 after 40 min. This may suggest that the tighter structure of the 1 kDa membrane limited the penetration of some contaminants into the membrane structure and favored surface deposition. However, the dominant fouling mechanism cannot be identified without additional resistance analysis or membrane surface characterization. Although such a layer reduces permeability, it may be easier to remove during regeneration than internal fouling associated with pore blockage.

A different pattern was observed for the 50 kDa membrane. Although this membrane had a higher MWCO than the 1, 5, and 15 kDa membranes, its relative permeate flux decreased from approximately 0.036 to approximately 0.008 after 40 min of filtration. This corresponds to a decrease of approximately 77% from the initial value. The result indicates that a higher MWCO did not automatically translate into more favorable short-term flux behavior. For the 50 kDa membrane, larger pores may have favored the penetration or partial deposition of colloidal particles, organic macromolecules, or fine aggregates within the membrane structure. This interpretation is consistent with the pronounced J/J_0_ decline observed for this membrane, but it should be treated as a hypothesis rather than as direct evidence of internal fouling. This phenomenon is particularly important in brewery wastewater treatment because the wastewater contains a mixture of contaminants with different sizes and aggregation tendencies.

A comparison of the curves for the ceramic membranes indicates that the most pronounced decrease in J/J_0_ occurred during the initial filtration period, i.e., within the first 5–10 min of operation. During this period, rapid pore constriction and/or initial deposit layer formation may have occurred. At a later stage, the rate of decline in relative permeate flux was lower, which may indicate a transition to more stable filtration through the growing cake layer. Such behavior is typical of wastewater with a high colloidal and organic load, for which the initial interaction between the feed and membrane surface determines subsequent transport behavior.

The RO membrane exhibited distinctly different behavior from the MF and UF membranes. Its J/J_0_ value was substantially higher than those of the ceramic membranes, at approximately 0.277 at the beginning of the process and approximately 0.221 after 40 min of filtration. The relative permeate flux therefore decreased by approximately 20%, and J/J_0_ remained practically stable after the first few minutes of operation. However, it should be emphasized that the RO membrane operated in a different treatment configuration from the MF and UF membranes because its feed was the permeate from the preceding UF stage. Thus, the stream fed to the RO membrane had already been largely depleted of suspended solids, colloids, and some macromolecular organic matter. The relatively stable J/J_0_ during RO is consistent with the beneficial role of feed pretreatment before final polishing. However, this comparison should be interpreted with caution because the RO membrane operated with a different membrane material, pressure, and feed quality than the ceramic MF and UF membranes.

The results indicate that membrane selection for brewery wastewater treatment must consider not only separation efficiency but also fouling susceptibility. Despite their larger pores, the MF membranes exhibited the lowest relative permeate fluxes, which limits their suitability as stand-alone treatment stages. The 5 and 15 kDa membranes exhibited more favorable transport properties, and the 15 kDa membrane provided the highest final J/J_0_ among the ceramic membranes. In contrast, despite its lower relative flux, the 1 kDa membrane may be advantageous in terms of organic contaminant separation efficiency, demonstrating the need to balance permeate quality and short-term flux behavior.

The results presented in Figure 4 show that membrane selection should consider not only permeate quality but also short-term flux behavior. The MF membranes exhibited the lowest relative permeate fluxes, whereas the 5 and 15 kDa membranes showed more favorable J/J_0_ values during the 40 min test. The 1 kDa membrane produced better organic contaminant removal, but at the expense of lower relative flux. The RO membrane showed the most stable short-term J/J_0_ profile; however, this result should not be directly compared with the ceramic membrane tests because RO was operated under different pressure conditions and with a pretreated UF permeate as feed. Overall, the observed trends suggest that pretreatment and membrane MWCO influence short-term hydraulic behavior, but the identification of specific fouling mechanisms would require additional experiments, including resistance analysis, blocking model fitting, surface imaging, and permeability recovery tests.

### 3.4. Changes in Particle Size Distribution After Successive Treatment Stages

**Figure 5:**
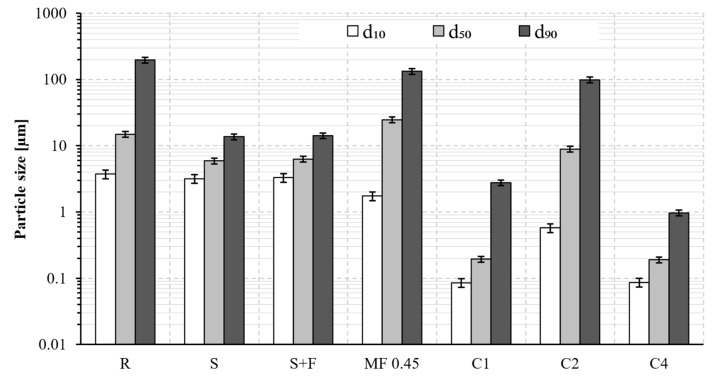
Particle size distribution in brewery wastewater samples after selected treatment stages, expressed as D10, D50, and D90 values. C1, C2, and C4 denote coagulation with 1, 2, and 4 mL PIX 113/L of wastewater, respectively.

Particle size distribution analysis indicated (Figure 5) that the successive brewery wastewater treatment stages removed different contaminant fractions. The raw wastewater exhibited a broad particle size distribution, with D10, D50, and D90 values of 3.7 µm, 14.9 µm, and 196.4 µm, respectively. The high D90 value indicated the presence of a substantial quantity of large suspended particles or loose aggregates, which are typical of brewery wastewater containing suspended biomass, cell fragments, proteins, polysaccharides, and colored organic compounds.

Sedimentation effectively removed the largest particles. Following this process, D90 decreased from 196.4 to 13.7 µm, whereas D50 decreased from 14.9 to 5.9 µm. Subsequent filtration through a 1 µm filter did not substantially alter the particle size distribution, as the D10, D50, and D90 values were 3.3 µm, 6.3 µm, and 14.2 µm, respectively. These results indicate that sedimentation and prefiltration effectively reduced the coarse fraction but did not completely remove the fine particles and colloids remaining in the liquid.

MF of wastewater after sedimentation using the 0.45 µm membrane without prior coagulation did not produce a clear narrowing of the particle size distribution. In the permeate from this process, D90 remained as high as 133.1 µm, and D50 increased to 24.7 µm. Thus, MF alone, without prior destabilization of the colloidal system, was insufficient for effectively removing the particles and aggregates present in brewery wastewater. This result indicates the need for additional feed pretreatment before membrane filtration.

Coagulation markedly altered the nature of the dispersion. At a dosage of 1 mL PIX 113/L of wastewater, the D10, D50, and D90 values were 0.1 µm, 0.2 µm, and 2.8 µm, respectively, indicating an effective reduction in the proportion of large particles. At a dosage of 2 mL PIX 113/L of wastewater, which was subsequently selected for further membrane processes, D10, D50, and D90 values of 0.6 µm, 8.9 µm, and 98.9 µm, respectively, were obtained. The broader particle size distribution in this sample can be attributed to the presence of flocs that were not completely separated during sedimentation and filtration. At the same time, this configuration produced favorable sample clarity and represented a compromise among treatment efficiency, sample stability, and the amount of coagulant added.

The highest coagulant dosage, 4 mL PIX 113/L of wastewater, resulted in the lowest D50 and D90 values, at 0.2 µm and 1.0 µm, respectively. However, this does not mean that this configuration was the most technologically favorable. Visual observations indicated that the sample treated with the highest coagulant dosage was darker and more turbid than the samples treated with lower dosages. This may indicate the presence of fine suspended matter originating from excess coagulant or its hydrolysis products. Therefore, selection of a coagulant dosage should not be based solely on minimizing D50 and D90 but should also account for sample clarity, organic contaminant removal efficiency, and reagent consumption.

The sample obtained after coagulation at a dosage of 2 mL PIX 113/L of wastewater and filtration through filter paper, which served as the feed for subsequent membrane processes, had D10, D50, and D90 values of 2.7 µm, 10.3 µm, and 20.0 µm, respectively. This distribution indicates the presence of micrometer-scale flocs formed during coagulation. However, the proportion of the largest particles was substantially lower than in the raw wastewater, supporting the suitability of coagulation and filtration as pretreatment for subsequent membrane separation.

**Figure 6:**
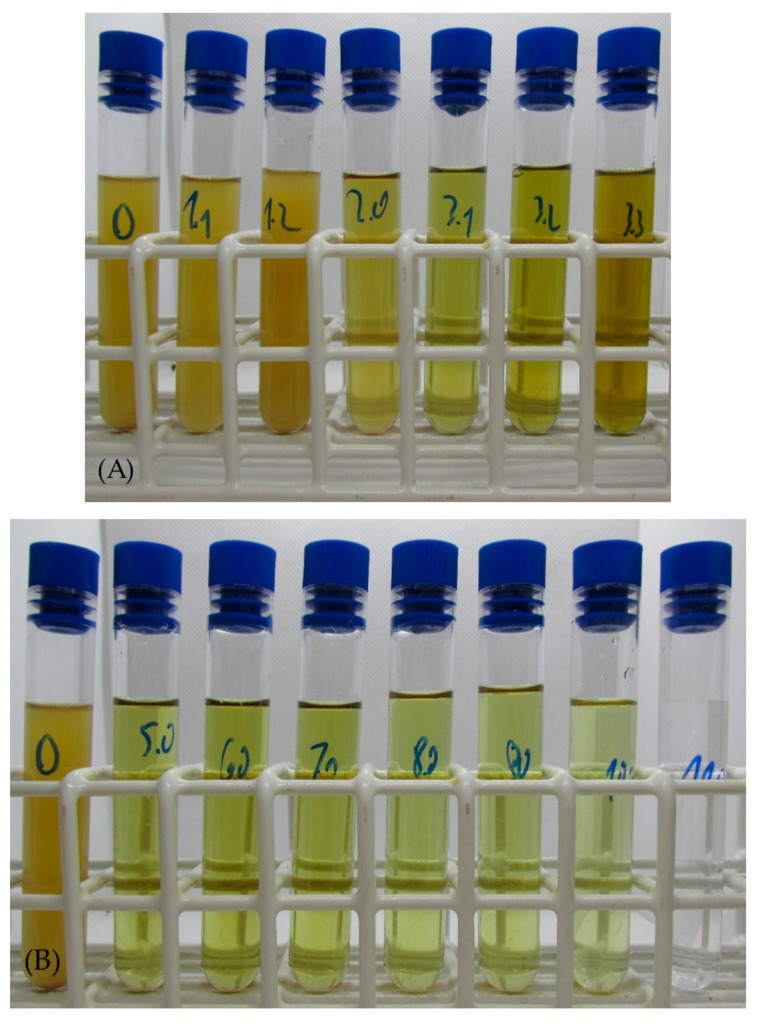
Visual changes in brewery wastewater samples after successive treatment stages: (**A**) samples before membrane filtration, including raw wastewater (R), sedimented wastewater (S), sedimented and prefiltered wastewater (S+F), MF 0.45, C1, C2, and C4; (**B**) raw wastewater and permeates obtained after membrane filtration preceded by coagulation, including C2+MF 0.45, C2+MF 0.14, C2+UF 50, C2+UF 15, C2+UF 5, C2+UF 1, and C2+UF 50+RO.

The permeates obtained after MF, UF, and fine UF/NF were visually clear (Figure 6) but retained a yellowish-green hue. DLS measurements revealed nanometer-scale fractions in these samples, with peaks occurring primarily at approximately 1–3 nm, 5–10 nm, and 20–200 nm. These fractions may correspond to dissolved organic matter, fine nanocolloids, and organic–inorganic complexes that were not completely retained by the porous membranes. High PDI values indicated the polydisperse nature of the permeates and thus the presence of a mixture of particles and structures of different sizes.

The persistent color of the permeates after MF, UF, and fine UF/NF indicates that some low-molecular-weight organic compounds, probably including chromophores responsible for color, passed through the porous membranes. Only the permeate obtained from the 50 kDa UF + RO system was colorless and completely transparent. For this sample, the DLS measurement was unreliable because of the very low signal intensity, indirectly suggesting the very low concentration of light-scattering particles. This result indicates that the final RO stage was necessary to obtain permeate with the highest optical clarity.

Thus, particle size distribution analysis was consistent with the sequential nature of contaminant removal from brewery wastewater. Sedimentation and prefiltration primarily removed the largest suspended particles; coagulation altered the colloidal system and prepared the feed for membrane filtration; and MF, UF, and fine UF/NF reduced the proportion of fine particles and part of the colloidal fraction. However, the porous membranes did not completely remove the nanometer-scale fraction and dissolved colored compounds. Only the combination of UF and RO produced a colorless and clear permeate, supporting the use of an integrated system for brewery wastewater treatment aimed at improving the quality of recovered water.

### 3.5. Characterization of Nanometer-Scale Fractions in Raw Wastewater, Coagulated Samples, and Membrane Permeates

**Figure 7:**
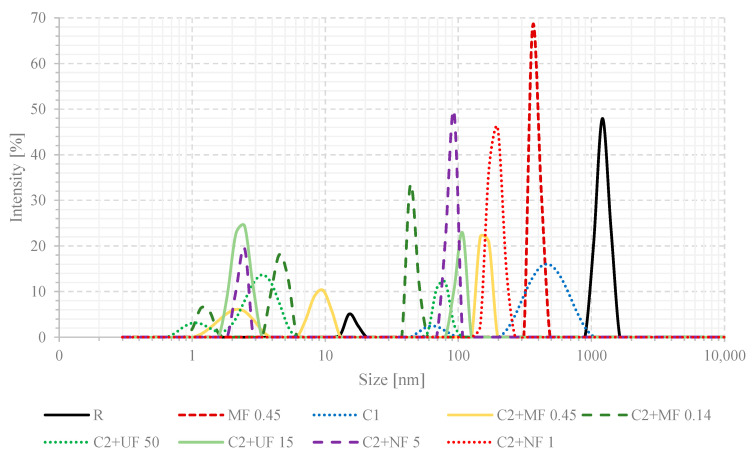
Dynamic light scattering (DLS) particle size distributions of raw brewery wastewater, coagulated samples, and permeates obtained after MF, UF, and fine ultrafiltration/nanofiltration (fine UF/NF) treatment.

DLS was used to supplement the characterization of the colloidal system by assessing nanometer-scale fractions that were not fully characterized by the D10, D50, and D90 values obtained using laser diffraction. The results presented in Figure 7 indicate that the raw wastewater, coagulated samples, and membrane permeates all exhibited heterogeneous, polydisperse particle size distributions. The presence of peaks ranging from several to several hundred nanometers indicated that despite the gradual removal of suspended solids and larger colloids, fine colloidal fractions, nanocolloids, and dissolved or partially aggregated organic constituents remained in the system.

The raw wastewater exhibited a dominant peak above 1000 nm, indicating the presence of large colloidal structures, organic suspended solids, and aggregates typical of high-strength brewery wastewater. This result is consistent with the previous characterization of the raw samples, which exhibited intense color, high turbidity, and large particles detected by laser diffraction. However, it should be emphasized that DLS results for such complex, highly turbid, and polydisperse samples should primarily be interpreted qualitatively. This technique is highly sensitive to larger particles, which may dominate the scattering signal and mask the contribution of smaller fractions.

The permeate obtained by MF of wastewater after sedimentation using the 0.45 µm membrane without prior coagulation exhibited a distinct peak in the range of several hundred nanometers. Thus, despite improving sample clarity, MF alone did not completely remove the fine colloidal fractions. This result was consistent with the limited effectiveness of membrane filtration after sedimentation for brewery wastewater with a high colloidal load. The absence of prior destabilization promoted the passage of fine, stable particles through the membrane or their persistence in the permeate as a result of excessive loading of the membrane surface.

Compared with the raw wastewater, the sample coagulated at a dosage of 1 mL PIX 113/L of wastewater exhibited a distribution shifted toward smaller sizes. The presence of a peak in the range of several hundred nanometers indicates that coagulation altered the colloidal system and partially removed larger aggregates but did not completely eliminate fine colloidal structures. This may result from the presence of coagulant hydrolysis products, fine flocs, or residual dissolved organic matter that retained the ability to scatter light.

The permeates obtained after coagulation at a dosage of 2 mL PIX 113/L of wastewater and filtration through the MF, UF, and fine UF/NF membranes exhibited distinctly different particle size distributions. The permeates from the C2+MF 0.45 and C2+MF 0.14 membranes contained fractions ranging in size from several tens to more than one hundred nanometers. This indicates that MF after coagulation effectively reduced the presence of larger particles but did not provide a complete barrier to the smallest colloidal and nanocolloidal fractions. Nevertheless, compared with direct MF without coagulation, the proportion of larger structures was favorably reduced, supporting the importance of coagulation as a feed pretreatment stage before subsequent membrane separation.

The permeates obtained after UF and fine UF/NF using membranes with lower MWCOs exhibited peaks ranging from several to several hundred nanometers. For the C2+UF 50 and C2+UF 15 membranes, nanometer-scale fractions were observed that may correspond to fine colloids, dissolved organic matter fragments, and organic–inorganic complexes. For the C2+UF 5 and C2+UF 1 samples, part of the signal shifted further toward smaller sizes, indicating more effective reduction of larger nanocolloids as membrane MWCO decreased. At the same time, the presence of a DLS signal in these samples indicates that the fine UF/NF membranes did not completely remove the smallest constituents present in the solution.

The results indicate that the permeates from the MF, UF, and fine UF/NF processes were not homogeneous systems. The presence of several nanometer-scale fractions indicates their polydisperse nature and the coexistence of different groups of constituents, including low-molecular-weight organic matter, nanocolloids, and organic–inorganic complexes. These results are consistent with the visual observations that the permeates from the porous membranes were clear but retained a yellowish-green hue. The persistent color may be associated with melanoidins and other chromophoric organic compounds that, because of their small size and chemical properties, can pass through MF, UF, and fine UF/NF membranes.

A comparison of all analyzed samples indicates the sequential nature of the transformations occurring during brewery wastewater treatment. The raw wastewater was dominated by large colloidal structures and suspended solids, whereas coagulation and successive membrane processes gradually reduced the proportion of larger particles and shifted the distribution toward nanometer-scale fractions. The porous membranes effectively removed suspended solids and part of the colloidal fraction but did not completely eliminate fine dissolved and nanocolloidal constituents. The DLS results therefore suggest that MF, UF, or fine UF/NF alone, even after coagulation, may be insufficient to completely remove the smallest fractions responsible for color and residual dissolved organic matter from brewery wastewater. Consequently, obtaining permeate with the highest clarity and quality requires a tighter separation barrier, such as RO, as the final polishing stage.

### 3.6. Changes in Zeta Potential During Brewery Wastewater Treatment

**Figure 8:**
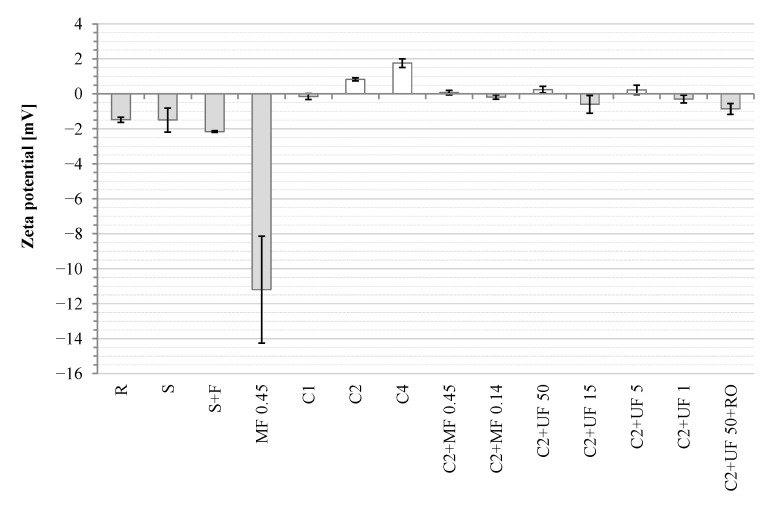
Changes in zeta potential of brewery wastewater samples after successive treatment stages.

Zeta potential analysis was used to assess changes in the colloidal stability of brewery wastewater after successive treatment stages (Figure 8). This parameter characterizes the surface charge of particles and colloids present in the system, thereby facilitating interpretation of the effectiveness of sedimentation, coagulation, and membrane filtration. Zeta potential values close to zero indicate limited electrostatic stability and a greater tendency of particles to aggregate, whereas more negative or positive values indicate stronger electrostatic repulsion between particles.

The raw brewery wastewater had a zeta potential of approximately −1.48 mV. This value was close to zero, indicating that the colloidal system was near its isoelectric point and prone to aggregation. This result is consistent with the preceding particle size distribution analysis, which revealed large aggregates in the raw wastewater. Sedimentation did not substantially change the zeta potential, as a value of approximately −1.50 mV was obtained for the sample after this stage. This indicates that sedimentation primarily removed larger, readily settleable particles but did not fundamentally alter the electrokinetic properties of the remaining colloidal fraction.

A similarly small change was observed after the combination of sedimentation and prefiltration. The zeta potential of the sample after the 1 µm filter was approximately −2.16 mV and therefore remained close to zero. Thus, like sedimentation, prefiltration was primarily mechanical and did not result in substantial destabilization or alteration of the particle surface charge. This indicates that sedimentation and coarse filtration reduce the suspended fraction but do not fundamentally modify the surface chemistry of the colloids present in the wastewater.

A different effect was observed after MF of the wastewater after sedimentation using the 0.45 µm membrane without prior coagulation. The zeta potential of this sample was approximately −11.20 mV and was therefore substantially more negative than those of the raw wastewater and the samples after sedimentation and prefiltration. At the same time, this measurement had the largest error, indicating substantial sample heterogeneity. Such a pronounced shift toward negative values may indicate that fine, more electrostatically stable colloidal fractions and dissolved organic matter remained in the permeate after MF following sedimentation. This result was consistent with the limited effectiveness of MF alone for brewery wastewater treatment without prior destabilization of the colloidal system.

Coagulation markedly altered the electrokinetic characteristics of the system. At the lowest coagulant dosage, designated C1, the zeta potential was approximately −0.15 mV and therefore practically equal to zero. This indicates effective neutralization of the particle surface charge and favorable conditions for aggregation. At the C2 dosage, the zeta potential became positive, at approximately +0.82 mV, whereas at the highest dosage, C4, it increased to approximately +1.76 mV. The gradual shift in zeta potential from negative to positive values with increasing coagulant dosage indicates a growing contribution of positively charged iron coagulant hydrolysis products and the possibility of partial reversal of the particle surface charge.

These results indicate that coagulation was the key stage in destabilizing the colloidal system. From the perspective of destabilization and aggregation, the most favorable zeta potential range was obtained at the C1 and C2 dosages, for which the values were closest to zero. At the C4 dosage, the positive zeta potential was higher, which may indicate excess coagulant and a partial change in the nature of the suspension. This is consistent with the previous visual observations and particle size distribution results, which indicated that despite reducing D50 and D90, the highest coagulant dosage was not necessarily the most technologically favorable configuration.

The C2 sample obtained after additional filtration through filter paper had a zeta potential of approximately +0.35 mV. This value was close to zero, indicating that a weakly charged fraction remained in the liquid after coagulation and filtration. This state favors further colloid removal by membrane processes because limited electrostatic stabilization reduces the ability of fine particles to remain in a stable dispersion.

The permeates obtained by membrane filtration after coagulation at the C2 dosage had zeta potentials close to zero. The zeta potential was approximately +0.07 mV after 0.45 µm MF, −0.18 mV after 0.14 µm MF, +0.24 mV after 50 kDa UF, −0.60 mV after 15 kDa UF, +0.22 mV after 5 kDa UF, and −0.30 mV after 1 kDa UF. These small differences indicate that stable, highly charged colloids no longer predominated in the permeates after coagulation and membrane filtration. Instead, the remaining fraction was likely composed of dissolved organic matter, nanocolloids, or fine organic–inorganic complexes with low surface charge.

It is also noteworthy that the use of membranes with different MWCOs did not produce a clear, monotonic shift in zeta potential. The values obtained for the permeates after MF, UF, and fine UF were within a narrow range from approximately −0.60 to +0.24 mV. Thus, membrane tightness alone was not the primary factor determining permeate zeta potential. Prior feed pretreatment by coagulation was more important because it reduced the colloidal stability of the system and removed a substantial proportion of the particles responsible for surface charge.

The final permeate obtained from the 50 kDa UF + RO system had a zeta potential of approximately −0.86 mV. This value was also close to zero, although slightly more negative than those of most permeates after MF and UF. It may be associated with the small quantity of low-molecular-weight organic compounds or ionic constituents remaining in the permeate after RO. Together with the observed complete clarity and absence of color, this result was consistent with highly effective removal of colloidal fractions and constituents responsible for turbidity.

In summary, zeta potential analysis was consistent with the sequential nature of the changes occurring during brewery wastewater treatment. Sedimentation and prefiltration primarily removed larger particles without substantially changing the electrokinetic properties of the system. MF after sedimentation without coagulation produced permeate containing more negatively charged and stable fractions, indicating the insufficient effectiveness of this approach as a stand-alone membrane treatment stage. Coagulation shifted the zeta potential toward values close to zero or slightly positive, indicating effective charge neutralization and colloid destabilization. Combining coagulation with membrane filtration produced permeates with zeta potentials close to zero, indicating a substantial reduction in the proportion of stable colloids. These results indicate that coagulation performs a key feed pretreatment function before subsequent membrane separation and that the final RO stage produces the permeate with the highest clarity.

The investigated process should be regarded as a short-term laboratory assessment of the role of individual separation barriers rather than as a ready-to-implement brewery wastewater treatment technology. Owing to the high biodegradable organic load of the wastewater, biological treatment would usually remain an important reference or preceding step in practical applications. The present study focused on permeate quality improvement by sedimentation, prefiltration, coagulation, ceramic membrane filtration, and RO polishing. Full-scale feasibility would require further assessment of biological treatment integration, volumetric recovery, mass balance, energy demand, chemical consumption, sludge and retentate management, and compliance with specific reuse or discharge criteria.

Based on the measured parameters, the UF-RO permeate represented the highest-quality stream obtained in this study, with COD, BOD_5_, and DOC reduced to 1033 mg O_2_/L, 468 mg O_2_/L, and 389 mg C/L, respectively. The corresponding residual chloride and nitrate concentrations, calculated from the measured raw-wastewater concentrations and overall removals, were approximately 9.8 mg/L and 2.1 mg/L, respectively. These results provide a partial quality-based indication of the reuse potential of the UF-RO permeate, but they do not constitute a formal compliance assessment for any specific reuse category because parameters such as microbiological quality, disinfection requirements, process water compatibility, and site-specific regulatory thresholds were outside the scope of this study.

## 4. Conclusions

The study demonstrated that high-strength brewery wastewater requires multistage treatment because sedimentation and prefiltration alone provided only limited reductions in COD, BOD_5_, and DOC.

Coagulation with PIX 113 substantially improved wastewater quality and reduced the load entering the membrane stage. A dosage of 2 mL/L was selected as a favorable compromise among the tested dosages, although it should not be interpreted as a universally optimal dose.

Among the treatment trains based on coagulation followed by ceramic membrane filtration, the train ending with the 1 kDa membrane produced the highest-quality permeate. The overall removals calculated relative to the raw wastewater were 78.2% for COD, 88.7% for BOD_5_, and 49.8% for DOC. These values reflect the cumulative effect of pretreatment and membrane filtration rather than the isolated rejection of the 1 kDa membrane.

The tested sedimentation–prefiltration–coagulation-50 kDa UF-RO train produced the greatest improvement in permeate quality among the investigated configurations. Overall removals of COD, BOD_5_, and DOC were 97.9%, 98.6%, and 94.0%, respectively, while the overall removals of chloride and nitrate ions were 92.5% and 68.5%, respectively.

The results indicate that ceramic membrane filtration and RO polishing can serve complementary functions in the treatment of brewery wastewater. Ceramic membranes mainly reduced suspended, colloidal, and macromolecular fractions, whereas RO substantially improved the removal of dissolved organic compounds and selected inorganic ions.

Consequently, RO should not be regarded as a default stage for every brewery wastewater treatment objective. A lower-intensity train based on coagulation and ceramic UF may be preferred when removal of suspended, colloidal, and biodegradable organic fractions is sufficient, whereas RO is justified when the required endpoint includes high DOC and color reduction, dissolved-ion removal, or high-quality water recovery.

The study should be interpreted as a short-term laboratory assessment of permeate quality improvement. Volumetric water recovery, mass balance, retentate and sludge management, microbiological quality, disinfection requirements, and compliance with specific reuse criteria were not assessed. Therefore, although the UF-RO train markedly decreased COD, BOD_5_, DOC, chloride, and nitrate concentrations, compliance with a defined reuse category cannot be confirmed from the present data set, and the direct suitability of the obtained permeate for water reuse requires further dedicated evaluation.

## Figures and Tables

**Table 1:** Characteristics of the membranes used in the study (based on manufacturer specifications [21,22,23,24,25]).

Process	Membrane Type	Designation	Material	pH Range	Maximum Pressure, MPa	Maximum Temperature, °C	Active Area, cm^2^
MF	Ceramic	0.45 µm	ZrO_2_-TiO_2_	0–14	0.4	350	56
MF	Ceramic	0.14 µm	ZrO_2_-TiO_2_	0–14	0.4	350	56
UF	Ceramic	50 kDa	ZrO_2_	0–14	0.4	350	56
UF	Ceramic	15 kDa	ZrO_2_	0–14	0.4	350	56
Fine UF/NF	Ceramic	5 kDa	TiO_2_	2–14	0.4	350	56
Fine UF/NF	Ceramic	1 kDa	TiO_2_	2–14	0.4	350	56
RO	Polymeric	X201	Aromatic polyamide	2–11	4.0	45	17.4

**Table 2:** Treatment configurations investigated in the study.

Configuration	Process Train	Comparison Objective
Configuration 1	Sedimentation—0.45 µm ceramic MF	Evaluation of the effectiveness of a simple sedimentation + MF system
Configuration 2	Sedimentation—1 µm prefiltration—PIX 113 coagulation—post-coagulation settling—qualitative filter paper filtration—MF/UF/fine UF membrane filtration	Evaluation of the effects of pretreatment and ceramic membrane pore size/MWCO
Configuration 3	Sedimentation—1 µm prefiltration—PIX 113 coagulation—post-coagulation settling—qualitative filter paper filtration—50 kDa UF—X201 RO	Evaluation of the effectiveness of an integrated UF + RO system

**Table 3:** Abbreviated designations used for wastewater samples and treatment configurations.

Designation	Full Description
R	Raw brewery wastewater.
S	Sample collected after sedimentation.
S+F	Wastewater subjected to sedimentation and prefiltration through a 1 µm filter.
MF 0.45	Permeate obtained by MF of wastewater after sedimentation using a 0.45 µm ceramic membrane without prior coagulation.
C1	Sample obtained after sedimentation, 1 µm prefiltration, coagulation with 1 mL PIX 113/L of wastewater, post-coagulation settling, and gravity filtration through qualitative medium-speed cellulose filter paper.
C2	Sample obtained after sedimentation, 1 µm prefiltration, coagulation with 2 mL PIX 113/L of wastewater, post-coagulation settling, and gravity filtration through qualitative medium-speed cellulose filter paper.
C4	Sample obtained after sedimentation, 1 µm prefiltration, coagulation with 4 mL PIX 113/L of wastewater, post-coagulation settling, and gravity filtration through qualitative medium-speed cellulose filter paper.
C2+membrane	General notation for membrane configurations preceded by C2 pretreatment; the second part of the designation identifies the membrane used.
C2+MF 0.45; C2+MF 0.14	Permeates obtained by MF using the 0.45 and 0.14 µm ceramic membranes, respectively, after C2 pretreatment.
C2+UF 50; C2+UF 15	Permeates obtained by UF using the 50 and 15 kDa ceramic membranes, respectively, after C2 pretreatment.
C2+UF 5; C2+UF 1	Permeates obtained using ceramic membranes with MWCOs of 5 and 1 kDa, respectively, after C2 pretreatment; these membranes are referred to in this study as fine UF/NF membranes.
C2+UF 50+RO	Final permeate obtained using the integrated system comprising C2 pretreatment, UF using the 50 kDa ceramic membrane, and RO.

**Table 4:** Characteristics of the raw brewery wastewater.

Parameter	Value
pH	4.36
Conductivity, mS/cm	1.6504
Chemical oxygen demand (COD), g O_2_/m^3^	48,230
Biochemical oxygen demand (BOD_5_), g O_2_/m^3^	34,160
Dissolved organic carbon (DOC), g C/m^3^	6492
Chloride, g/m^3^	130
Fluoride, g/m^3^	<LOQ
Nitrate, g/m^3^	6.7
Phosphate, g/m^3^	34.4
Total phosphorus, g/m^3^	52.8
Total nitrogen, g/m^3^	40.6
Ammonium, g/m^3^	13.3
Dry residue, g/m^3^	10,400
Suspended solids, g/m^3^	1284
Dissolved solids, g/m^3^	9116
Mineral solids, g/m^3^	942
Volatile solids, g/m^3^	9444

LOQ, limit of quantification.

**Table 5:** Observed membrane rejection calculated relative to the feed pretreated with 2 mL PIX 113/L (C2) entering the membrane stage.

Membrane	COD, %	BOD_5_, %	DOC, %
MF 0.45 µm	7.0	8.5	~0 ^a^
MF 0.14 µm	12.8	30.7	~0 ^a^
UF 50 kDa	25.5	48.8	7.8
UF 15 kDa	30.2	56.6	8.5
Fine UF/NF 5 kDa	37.3	64.8	19.4
Fine UF/NF 1 kDa	53.7	71.4	26.7

^a^ Calculated DOC rejection was below the analytical uncertainty limit and is therefore reported as approximately zero.

## Data Availability

The original contributions presented in this study are included in the article. Further inquiries can be directed to the corresponding author.
